# Absence of Nonclassical Monocytes in Hemolytic Patients: Free Hb and NO-Mediated Mechanism

**DOI:** 10.1155/2019/1409383

**Published:** 2019-03-27

**Authors:** Rashi Singhal, Deepak K. Rathore, Teena Bhakuni, Tulika Seth, Prasenjit Guchhait

**Affiliations:** ^1^National Capital Region Biotech Science Cluster, Disease Biology Laboratory, Regional Centre for Biotechnology, Faridabad, India; ^2^National Capital Region Biotech Science Cluster, Translational Health Science and Technology Institute, Faridabad, India; ^3^All India Institute of Medical Sciences, Department of Hematology, New Delhi, India

## Abstract

In a recent work, we have described the kinetics among the monocyte subsets in the peripheral blood of hemolytic patients including paroxysmal nocturnal hemoglobinuria (PNH) and sickle cell disease (SCD). After engulfing Hb-activated platelets, classical monocytes (CD14^+^CD16^−^) significantly transformed into highly inflammatory (CD14^+^CD16^hi^) subsets *in vitro*. An estimated 40% of total circulating monocytes in PNH and 70% in SCD patients existed as CD14^+^CD16^hi^ subsets. In this study, we show that the nonclassical (CD14^dim^CD16^+^) monocyte subsets are nearly absent in patients with PNH or SCD, compared to 10-12% cells in healthy individuals. In mechanism, we have described the unique role of both free Hb and nitric oxide (NO) in reducing number of nonclassical subsets more than classical monocytes. After engulfing Hb-activated platelets, the monocytes including nonclassical subsets acquired rapid cell death within 12 h *in vitro*. Further, the treatment to monocytes either with the secretome of Hb-activated platelets containing NO and free Hb or purified free Hb along with GSNO (a physiological NO donor) enhanced rapid cell death. Besides, our data from both PNH and SCD patients exhibited a direct correlation between intracellular NO and cell death marker 7AAD in monocytes from the peripheral blood. Our data together suggest that due to the immune surveillance nature, the nonclassical or patrolling monocytes are encountered frequently by Hb-activated platelets, free Hb, and NO in the circulation of hemolytic patients and are predisposed to die rapidly.

## 1. Introduction

In healthy individuals, peripheral blood monocytes are categorized mainly into three subsets by surface expression of CD14, a coreceptor for toll-like receptor 4 (TLR4) and CD16, a low affinity Fc*γ* receptor. These subsets include classical CD14^++^CD16^+^ monocytes, which have high expression of CD14; nonclassical CD14^+^CD16^++^ monocytes, which have high expression of CD16 and very low levels of CD14; and intermediate/inflammatory CD14^++^CD16^+^ monocytes, which coexpress CD16 together with high levels of CD14 [1]. Monocytes are highly plastic and heterogeneous and change their functional phenotype in response to environmental stimulation [2–5]. Classical monocytes are mainly responsible for performing phagocytosis and antigen presentation. They also differentiate into other cell types such as dendritic cells (DCs) and macrophages [6]. Inflammatory monocytes selectively traffic to the sites of inflammation, produce inflammatory cytokines, and contribute to local and systemic inflammation. Nonclassical monocytes are also known as patrolling monocytes and have been proposed to act as custodians of the vasculature by patrolling endothelial cell integrity in LFA-1-dependent fashion [7]. They patrol on the blood vessel wall and remove the dead particles or debris and hence contribute in the resolution of inflammation. A study by Cros et al. showed that human CD14^dim^ monocytes patrol and sense nucleic acids and viruses via TLR7 and TLR8 receptors [5]. In healthy adult individuals, the frequency of classical monocytes varies from 80 to 90% of total monocytes. On the other hand, nonclassical monocytes constitute about 8-10%, whereas neonates have a low number of nonclassical monocytes [8, 9]. The phenotype and function of monocyte subsets change extensively in inflammatory disease conditions. It has been reported that the CD14^+^CD16^+^ cell population was expanded significantly during inflammatory and infectious disease conditions [10]. Extensive studies thus suggested that the microenvironment directly regulates the heterogeneity of monocytes and macrophages [11–13].

Hemolytic diseases are characterized by an abnormal breakdown of red blood cells and release of hemoglobin (Hb) in extracellular fluid. The cell-free Hb contributes to significant cytotoxic effects and deregulates several cellular functions [14]. One of our recent studies has described the effects of free Hb on platelet functions. The Hb binding to a platelet surface potentiated its activation in a concentration-dependentmanner. An estimated 40-50% of total circulating platelets in paroxysmal nocturnal hemoglobinuria (PNH) [15] and sickle cell disease (SCD) [16] patients existed in an activation state and were mostly Hb-bound. Activated platelets release several bioactive molecules including cytokines, chemokines, and nitric oxide (NO) that play a significant role in modulating the function of other immune cells [17, 18]. Several studies suggest that activated platelets and their secretome encounter very frequently phagocytic cells such as monocytes and macrophage and alter their functions in several disease conditions including hemolytic disorders [19–25], although previous studies have shown that free Hb scavenges NO and thereby regulates the bioavailability of NO, affecting several cellular functions including vasodilatation and platelet activation in hemolytic disease conditions [14, 26, 27]. Studies have shown that NO released from activated platelets inhibits further platelet activation [18]. NO plays an important role in the modulation of leukocytes' functions [28]. Besides, NO also induces apoptosis or necrosis and promotes cell death [29–33].

While investigating the dynamic changes in monocyte subsets under a hemolytic environment, we observed a significant reduction in nonclassical monocyte subsets in patients with either PNH or SCD. In a mechanism, we describe in this study the crucial role of free Hb along with NO, which is secreted in a large amount from Hb-activated platelets, in the promotion of monocyte death *in vitro*. Besides, our study also describes the direct correlation between intracellular NO and apoptosis or cell death in monocytes from the peripheral blood of hemolytic patients with either PNH or SCD.

## 2. Methods

### 2.1. Major Reagents

Fluorescent-conjugated antihuman monoclonal antibodies for CD45 (HI30), CD11c (B-ly6), CD14 (M5E2), CD16 (3G8), and 7-AAD were purchased from BD Biosciences (San Jose, USA). Human CCR2 (K036C2) and CX3CR1 (K0124E1) antibodies were procured from BioLegend (San Diego, USA). Antihuman hemoglobin-*β* was purchased from Santa Cruz Biotechnology (Santa Cruz, CA, USA), and hemoglobin A and bovine serum albumin (BSA) were acquired from Sigma-Aldrich (St. Louis, USA).

### 2.2. Sample Collection

We recruited 10 patients with type III paroxysmal nocturnal hemoglobinuria (PNH) as confirmed by the complete absence of GPI-anchoring proteins CD55 and CD59 on red blood cells and also recruited 10 patients with sickle cell disease (SCD) diagnosed with HbS homozygosity by HPLC analysis. We also recruited 20 healthy volunteers (Suppl Table 1). All the patients gave their written consent according to the recommendations of the declaration of Helsinki. A peripheral blood sample (5-8 ml) was collected in vacutainers containing an acid-citrate-dextrose (ACD) anticoagulant for following assays.

### 2.3. Flow Cytometry

Immunophenotyping was performed using leukocytes isolated from the above patients and healthy individuals. The PBMCs were labelled with antihuman antibodies for CD14, CD16, CD45, CD11c, CCR2, and CX3CR1 and acquired using a flow cytometer (FACS Verse/FACS Canto-II/, BD Biosciences, San Jose, CA, USA) and analyzed using FlowJo software vX.0.6 (Treestar, Ashland, OR, USA). The monocyte subsets were sorted using fluorescence-activated cell sorting on a FACS Aria III cell sorter (BD Biosciences, San Jose, CA, USA) to obtain nonclassical (CD14^dim^CD16^+^) and classical (CD14^+^CD16^−^) subsets using anti-CD14 FITC, CD11c V450, and CD16 PECy7 monoclonal antibodies as mentioned in our previous work [34]. Cells were sorted at 70 psi and collected in tubes containing RPMI-1640 medium with excess FBS to maintain cell viability. The purity of sorted cells was typically 98% in the postsort analysis.

### 2.4. Preparation of Hb-Activated Platelet

Platelet-rich plasma (PRP) was obtained from the whole blood collected from healthy individuals by centrifugation at 500 rpm for 15 min. PRP was further centrifuged for 12 min to obtain the platelet pellet, which was resuspended in calcium-free tyrode HEPES buffer (126 mM NaCl, 2.7 mM KCl, 1 mM MgCl_2_, 0.38 mM NaH_2_PO_4_, 5.6 mM dextrose, 6.2 mM sodium HEPES, 8.8 mM HEPES-free acid, 0.1% bovine serum albumin (BSA), and pH 6.5) for wash. Washed platelets were isolated through Sepharose 2B (Sigma-Aldrich) gel filtration column for further use. To obtain the Hb-activated platelets, washed platelets were incubated with HbA at a concentration of 3.0 *μ*M (an *in vivo* plasma concentration in patients with either PNH or SCD [15, 16] and a concentration inducing *in vitro* platelet activation [15]) for 30 min at 37°C. Washed platelets were incubated with monocytes in a ratio of ~1 : 100 as previously mentioned [34].

### 2.5. In Vitro Stimulation of Monocytes

Peripheral blood mononuclear cells (PBMCs) were isolated from leukopacks obtained from the blood bank of AIIMS, New Delhi, by density gradient centrifugation using Lymphoprep (Axis Shield PoC AS, Oslo, Norway). PBMCs were cultured at 37°C for 2 h using complete RPMI 1640 medium (10% fetal bovine serum and 100 IU/ml penicillin-streptomycin procured from Sigma-Aldrich, St. Louis, and Gibco-Invitrogen, San Diego, CA, USA, respectively) and adherent monocytes collected and washed with phosphate-buffered saline (PBS). Monocytes were treated with HbA, HbA-activated platelets, thrombospondin, collagen, or GSNO. At various time points such as 0, 2, 12, 24, and 48 h, monocytes were collected and incubated for 10 min with ice-cold PBS containing 5 mM EDTA and subsequently labelled for flow immunophenotyping. As mentioned in Section 2.4, 3.0 *μ*M HbA and 200 *μ*M GSNO were used for all *in vitro* experiments.

### 2.6. S-Nitrosoglutathione (GSNO) Preparation

S-Nitrosoglutathione (GSNO) was prepared freshly before each experiment. 1.54 g of glutathione was dissolved in an acidic solution of 620 *μ*l of concentrated HCl in 5.9 ml H_2_O. NaNO_2_ (0.346 g/ml) was added into the above solution dropwise at night. pH was adjusted to 7.5 using 95% NaOH.

### 2.7. 2,3-Diaminonaphthalene (DAN) Assay

Naphthotriazole, which was formed by the reaction of DAN and NO, was quantified using fluorescence spectroscopy [35]. Supernatants from platelets treated with Hb (3.0 *μ*M) or thrombin (1 U/ml) were aliquoted into two equal parts; one part was treated with DAN (150 *μ*M), and the other part was treated with DAN (150 *μ*M) and copper acetate solution (150 *μ*M). The reaction mixture was incubated for 30 min at night, and the reaction volume was adjusted to a final volume of 200 *μ*l by adding 0.1 M NaOH. The OD was taken at an excitation wavelength of 375 nm and an emission wavelength of 450 nm using a Spectramax plate reader M5 (Molecular Devices, USA). NO release was quantified using a standard curve of NaNO_2_ [36].

### 2.8. Statistical Analysis

The experimental data are presented as mean ± standard error (SEM). Each experiment was performed at least 3 times. Statistical analysis was performed using a paired *t*-test between two treatments, and the comparison between two groups was performed using the Mann–Whitney *U* test. Graph Pad Prism 5.0 software was used for experimental data analysis. Values of *P* < 0.05 were considered to be statistically significant.

### 2.9. Study Approval

Ethical approval was obtained from the Institutional Ethics Committee for Human Research of the Regional Centre for Biotechnology (RCB, reference No. RCB-IEC-H-2) and All India Institute of Medical Sciences (AIIMS, reference No. IEC-NP-412/2013), New Delhi, India. Informed consent was provided according to the recommendations of the declaration of Helsinki.

## 3. Results

### 3.1. The Absence of Nonclassical Monocyte Subsets in Patients with Either PNH or SCD

**Figure 1:**
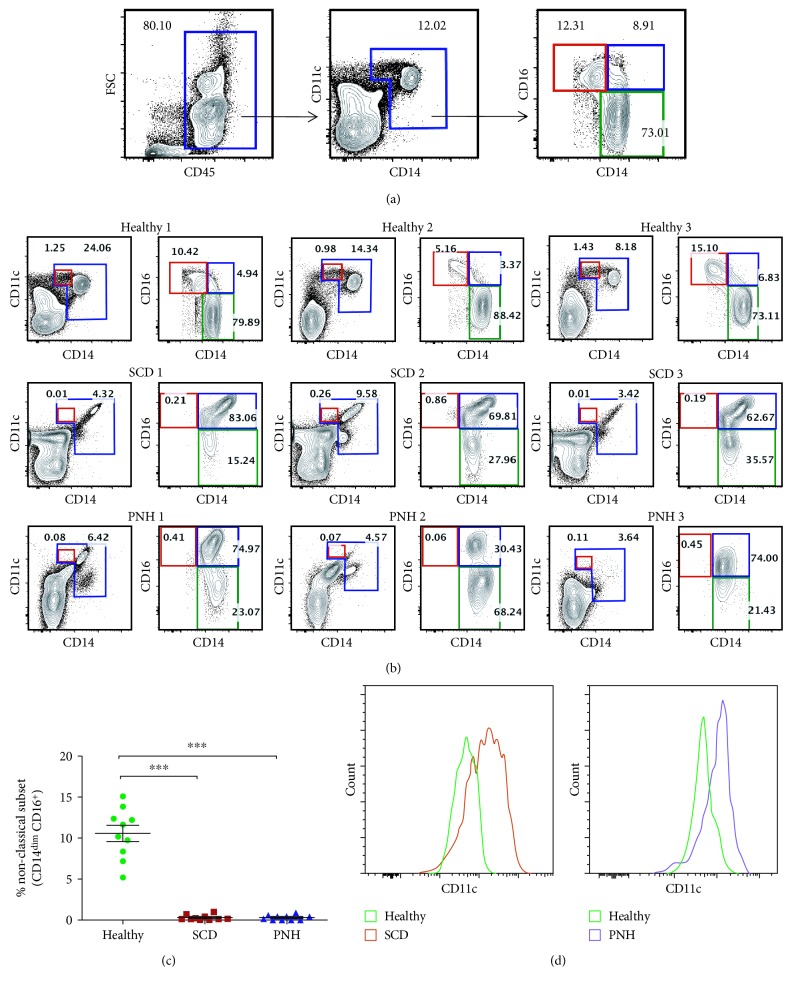
The absence of nonclassical monocyte subset in SCD and PNH patients. (a) Using flow cytometry, total leukocytes were gated as CD45^+^ and monocytes were gated as CD14^+^CD11c^+^ cells. Monocyte subsets were identified as CD14^dim^CD16^+^ nonclassical (red gated), CD14^+^CD16^+^ intermediate (blue gated), and CD14^+^CD16^−^ classical (green gated). (b) Representative flow cytometry plots of 3 individuals each from healthy, SCD, and PNH patients showed profiles of the nonclassical subset (red gate on both CD14 vs. CD11c plot and CD14 vs. CD16 plot). (c) Scattered dot plot showing frequencies of nonclassical monocyte subsets in healthy, SCD, and PNH patients (*n* = 10 each). Each dot represents percent-positive cells for an individual. The Mann–Whitney *U* test was used for the comparison between the groups (^∗∗∗^ *P* < 0.0001). (d) Representative histogram plots showing the expression of CD11c on monocytes of SCD, PNH, and healthy individuals.

We have assessed the phenotype of monocyte subsets in patients with SCD or PNH (*n* = 10 each) and compared with the healthy individuals (*n* = 20). Patients of both types exhibited less numbers of monocytes than healthy controls (Suppl Table S1). Monocytes were gated as CD11c- and CD14- positive cells, and three subsets of monocytes were identified accordingly: classical (CD14^+^CD16^−^), intermediate (CD14^+^CD16^+^), and nonclassical (CD14^dim^CD16^+^) (Figure 1(a)). In a recent work, we have reported a significant increase in intermediate monocyte subsets and a decrease in classical monocyte subsets in both PNH and SCD patients [34]. Further, our current study depicts a significant alteration in nonclassical monocyte subsets (CD14^dim^CD11c^high^). Our data show almost nil or very less (an estimated <0.5%) nonclassical monocytes (CD14^dim^CD16^+^) in the peripheral blood of patients with either PNH or SCD, compared to 10% cells in the healthy individuals (Figures 1(b) and 1(c)). We also observed a high expression of CD11c on monocytes of PNH and SCD patients in comparison to healthy individuals (Figure 1(d)), which can be attributed to high inflammation in hemolytic disease conditions.

We further characterized a monocyte subset using CX3CR1 and CCR2 as alternative markers for monocyte subsets and found negligible frequency of CD14^dim^CX3CR1^high^ cells which can be ascribed as a nonclassical subset in PNH patients (Suppl Fig 1A). Furthermore, our data confirmed the expression of CCR2 (Suppl Fig S1B) and CX3CR1 (Suppl Fig S1C) on classical, intermediate, and nonclassical monocyte subsets in healthy individuals. Interestingly, we did not observe a difference in the expression of CX3CR1 on classical versus nonclassical monocyte in PNH patients (Suppl Fig S1C), which is reported to be high on intermediate and nonclassical monocyte subsets. This may be due to the transformation of classical monocytes into an inflammatory subset as reported by us previously in hemolytic disease conditions [32].

### 3.2. Significant Reduction in Nonclassical Monocyte Subset upon Incubation with Hb-Activated Platelets In Vitro

**Figure 2:**
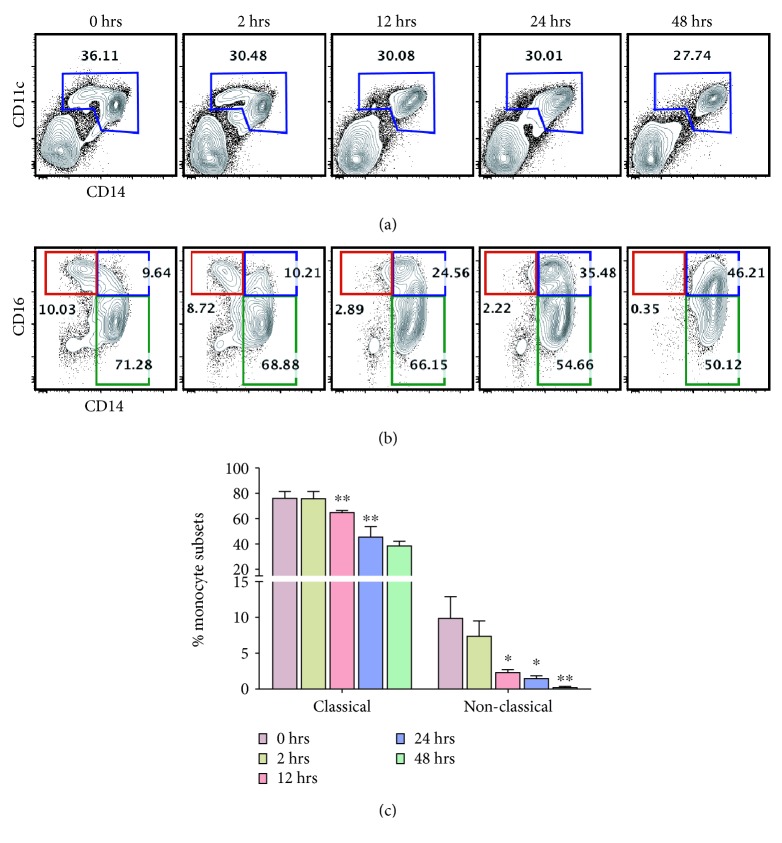
Dynamic changes in nonclassical monocytes after incubation with Hb-activated platelets *in vitro*. Monocytes from healthy individuals were incubated with Hb-activated platelets (0-48 h). After incubation with Hb-activated platelets, cells were harvested and processed for surface staining. (a) FACS plots showing the percentage of monocytes (CD11c^+^CD14^+^) at different time points and (b) percentage of nonclassical (red gated), intermediate (blue), and classical (green) monocyte subsets at 0, 2, 12, 24, and 48 h. (c) A bar diagram showing the percentage of nonclassical and classical monocyte subsets at different time points. Data are the mean ± SEM from three different experiments. The statistical significance was calculated using a paired *t*-test. ^∗^ *P* < 0.05, ^∗∗^ *P* < 0.01, and ^∗∗∗^ *P* < 0.001 compared to monocytes at 0 h.

In our recent study, we have reported a high percentage of circulating monocytes positive for intracellular Hb and platelets in PNH and SCD patients. *In vitro*, the classical monocytes (CD14^+^CD16^−^) were transformed into an inflammatory subset (CD14^+^CD16^+^) upon engulfment of Hb-activated platelets [34]. Our current data demonstrate that upon incubation with Hb-activated platelets, the total monocyte population (CD14^+^CD11c^+^) was decreased, (an estimated 10%) in 48 h *in vitro* (Figure 2(a)). More specifically, the number of nonclassical monocytes (CD14^dim^CD16^+^) was decreased rapidly and completely abolished within 48 h (Figures 2(b) and 2(c)).

### 3.3. Rapid Decrease in Monocyte Population upon Incubation with Free Hb and GSNO In Vitro

**Figure 3:**
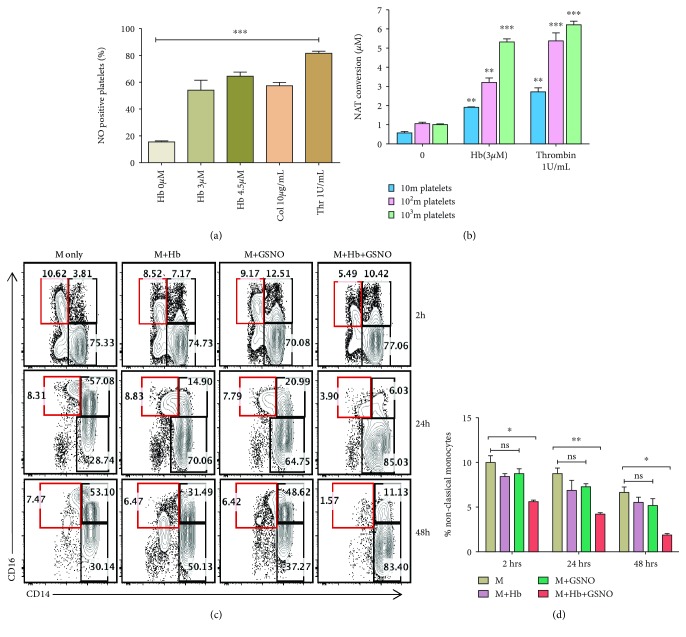
Effect of Hb and NO on monocytes. Washed platelets isolated from healthy individuals were stimulated with Hb, collagen, or thrombin for 30 minutes at 37°C with gentle rotation and further incubated with DAF-FM-DA dye for measuring NO levels using flow cytometry. (a) Data represent mean ± SEM from three different experiments showing the percentage of NO-positive platelets after incubation with the above agonists. ^∗∗∗^ *P* < 0.001, analyzed using a paired *t*-test. (b) DAN assay was performed to quantify nitric oxide release from platelets. DAN (2,3-diaminonaphthalene) reacts with nitric oxide (NO) to form fluorescent napthotriazole (NAT), which was quantified by fluorescence spectroscopy with an excitation at 375 nm and emission at 450 nm. NO was quantified from the supernatant of platelets incubated with either Hb (3 *μ*M) or thrombin (1 U/ml). Thrombin was used as a positive control. GSNO was used to prepare the standard curve for the quantification of NO release. Data are the mean ± SEM from 3 experiments. ^∗∗^ *P* < 0.01 and ^∗∗∗^ *P* < 0.001 compared to unstimulated platelets. Hb (0 *μ*M) was analyzed using one way analysis of variance with a Bonferroni post hoc test. (c) Monocytes isolated from the peripheral blood of healthy individuals were treated with either media alone, Hb, GSNO, or Hb+GSNO for 2, 24, and 48 h. After incubation, cells were harvested and processed for surface staining. Representative FACS plots from 3 independent experiments showing the percentage of CD14^dim^CD16^+^ nonclassical monocytes (red gated). (d) A bar diagram showing the percentage of nonclassical monocyte subsets at different time points. Data are the mean ± SEM from three different experiments. The statistical significance was calculated using a paired *t*-test. ^∗^ *P* < 0.05 and ^∗∗^ *P* < 0.01; ns = nonsignificant.

In order to investigate the molecular mechanism for the rapid decrease in the monocyte population in the presence of Hb-activated platelets (where platelets were activated by free Hb), we observed that the nitric oxide (NO), which is secreted in a vast amount by activated platelets (Figures 3(a) and 3(b)), along with free Hb promoted the significant death of monocytes. We used S-nitrosoglutathione (GSNO) as a physiological nitric oxide donor. GSNO decomposes through homolytic cleavage to liberate nitric oxide (NO) and the corresponding disulphide. GSNO can be synthesized chemically by the reaction of reduced glutathione with sodium nitrate in acidic condition. An estimated 3-4 *μ*M NO was produced by 100 million platelets, which is similar to a concentration released by 200 *μ*M GSNO, a physiological NO donor (Figure 3(b)). After incubating with both GSNO (200 *μ*M) and purified Hb (3 *μ*M), a significant decrease in a nonclassical monocyte count was observed *in vitro* (Figures 3(c) and 3(d)) indicating a possible role of circulating free Hb and NO in monocytopenia in hemolytic patients.

### 3.4. Hb and GSNO-Induced Rapid Death of Nonclassical Monocytes In Vitro

**Figure 4:**
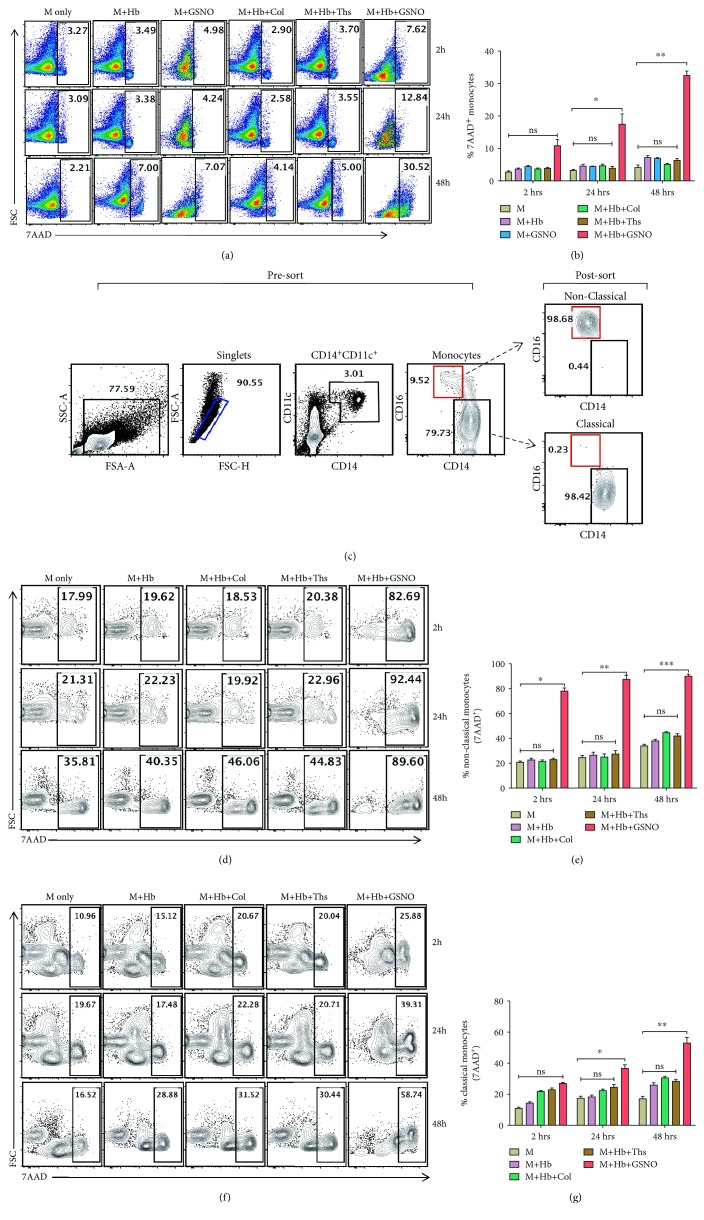
Hb and NO mediate the death of nonclassical monocytes. Total monocytes or separately nonclassical and classical monocytes from healthy individuals were incubated with media alone, Hb, Hb+collagen, Hb+thrombospondin, and Hb+GSNO for 2, 24, and 48 h. After incubation, cells were harvested and processed for 7-AAD staining to assess cell death using flow cytometry. (a) Representative FACS plots from 3 independent experiments showing the percentage of 7-AAD-positive monocytes. (b) A bar diagram showing the percentage of 7-AAD-positive monocytes at different time points. Data are the mean ± SEM from three different experiments. The statistical significance was calculated using a paired *t*-test. ^∗^ *P* < 0.05 and ^∗∗^ *P* < 0.01; ns = nonsignificant. (c) FACS sorting strategy of monocyte subsets from PBMCs. Cells were gated based on size (FSC-A vs. SSC-A), and doublet discrimination (FSC-H vs. FSC-A) was performed. Monocytes were gated as CD14^+^CD11c^+^ and further sorted based on CD14 and CD16 markers. After incubation with different treatments, monocytes were processed for 7-AAD staining. FACS plots (d and f) and bar diagram (e and g) showing the percentage of 7-AAD-positive nonclassical (d and e) and classical (f and g) monocytes, respectively. Data are the mean ± SEM from three different experiments. The statistical significance was calculated using a paired *t*-test. ^∗^ *P* < 0.05, ^∗∗^ *P* < 0.01, and ^∗∗∗^ *P* < 0.001; ns = nonsignificant. Hb+GSNO treatment to nonclassical monocytes significantly increased cell death.

We assessed the monocyte death after incubation with free Hb and GSNO *in vitro*. Our flow cytometry data showed a gradual increase in the number of 7-AAD^+^ monocytes at 2, 24, and 48 h (Figures 4(a) and 4(b)). We have gated nonmonocytic cells (other cell types) and analyzed 7-AAD. Hb+GSNO treatment showed a very less effect on nonmonocytic cells compared to that on monocytes (Suppl Fig S2). Also, we observed that nonclassical monocytes (CD14^dim^CD16^++^) from healthy individual PBMCs (Figure 4(c)) displayed a rapid increase in 7-AAD staining upon incubation with free Hb (3 *μ*M) and GSNO (200 *μ*M) (Figures 4(d) and 4(e)) compared to a classical subset (Figures 4(f) and 4(g)), confirming further the role of both molecules together predisposing the particular monocyte subsets towards cell death under a hemolytic environment.

### 3.5. NO-Positive Monocytes Display Increase Cell Death Markers in Patients with PNH or SCD

**Figure 5:**
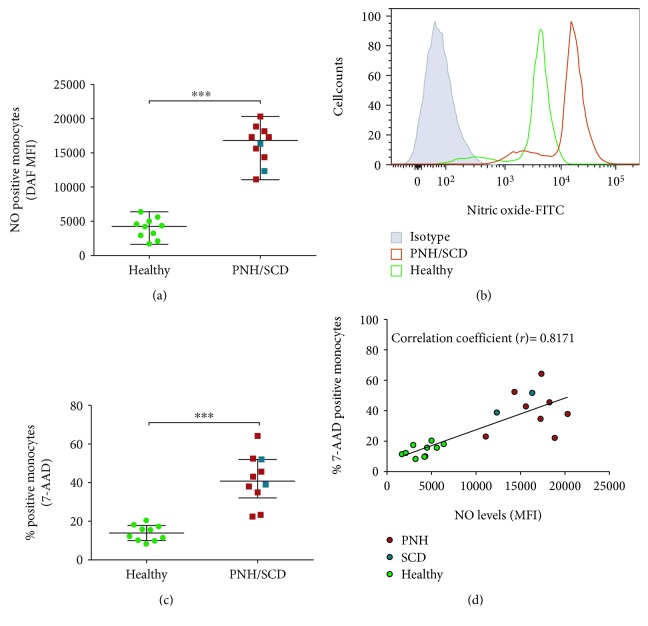
High NO level in monocytes of PNH/SCD patients correlates with 7AAD positivity. Monocytes from PNH (*n* = 8)/SCD (*n* = 2) patients (the same patients mentioned in Figure 1) and healthy individuals (*n* = 10) were stained with DAF-FM-DA dye for measuring NO levels or 7-AAD dye for assessing cell death using flow cytometry. (a) Scatter dotplot showing expression levels of NO. (b) A representative histogram showing the mean fluorescence intensity differences in PNH/SCD patients and healthy individuals. (c) A dotplot showing the percentage of 7-AAD-positive cells from PNH/SCD patients and healthy individuals. (d) A correlation plot showing the positive correlation between NO levels and cell death of monocytes from PNH/SCD patients. Each dot represents percent-positive cells for an individual. The Mann–Whitney *U* test was used for the comparison between the groups. ^∗∗∗^ *P* < 0.0001.

We have assessed the correlation between intracellular NO and death of monocytes in patients. Our data exhibited a high level of NO in monocytes from the peripheral blood of patients with either PNH or SCD (Figures 5(a) and 5(b)). Also, these patients' monocytes were positive for cell death marker 7-AAD (Figure 5(c)). The intracellular NO levels exhibited a direct correlation (*r* = ~0.8171) with 7AAD on monocytes isolated from patients with either of the above hemolytic disorders (Figure 5(d)).

## 4. Discussion

Monocytes are highly plastic and heterogeneous cells that change their phenotypes and functions with respect to environmental stimulants. These short-lived mononuclear cells are continuously replaced throughout life from a common committed progenitor, and their subset distribution and number are largely influenced by the microenvironment including hemolytic disease conditions [20, 24, 34]. In human, monocytes originate from the bone marrow, and mainly, the classical monocytes remain in circulation for 24 hours, whereas intermediate and nonclassical monocytes have a longer lifespan of 4 and 7 days, respectively [37].

Recently, we have described that the phenotype and function of monocytes were altered significantly under a hemolytic environment. Our study has shown the significant increase in an inflammatory monocyte subset in patients with PNH and SCD with intravascular hemolysis. An estimated 40% of total circulating monocytes in PNH and 70% in SCD patients existed as highly inflammatory (CD14^+^CD16^high^TNF-*α* ^+^) monocyte subsets. In a mechanism, we have described that after engulfing Hb-activated platelets, classical monocytes (CD14^+^CD16^−^) were significantly transformed into inflammatory (CD14^+^CD16^high^TNF-*α* ^+^) subsets. On the other hand, the classical monocytes exhibited anti-inflammatory phenotypes (CD14^**+**^CD16^low^IL-10^**+**^) after ingesting only free Hb [34]. The study described a clear heterogeneity in phenotype and function of monocytes when engulfed only Hb or both Hb and platelets together.

While investigating further the detail of monocyte subsets in hemolytic patients, we observed almost nil or very less (<0.5%) nonclassical monocytes (CD14^dim^CD16^+^) in patients with either PNH or SCD, compared to an 8-12% population in healthy individuals [8, 9]. The unique ability of nonclassical monocytes to actively patrol the vasculature and remove the dead particles or debris contributes in the resolution of inflammation. These monocyte subsets have been proposed to act as custodians of vasculature by patrolling endothelial cell integrity in an LFA-1-dependent fashion [7]. The CD14^dim^CD16^+^ monocyte subsets patrol and sense nucleic acids and viruses via TLR7 and TLR8 receptors [5]. Due to the immune surveillance nature, these nonclassical or patrolling monocytes are encountered frequently by pathogens, apoptotic cell debris, and other blood components. We investigated the mechanism for depletion of these nonclassical monocyte (CD14^dim^CD16^+^) subsets in patients with intravascular hemolysis in PNH and SCD. We observed that the number of nonclassical monocytes was decreased rapidly from 12 h after the engulfment of Hb-activated platelet and was completely abolished by 48 h *in vitro*. It could be possibly due to the transformation of this subset CD14^dim^CD16^+^into another subset such as inflammatory monocytes or due to increasing cell death after encountering Hb-activated platelets. Our data demonstrated that upon incubation with Hb-activated platelets, the CD14^+^CD11c^+^ monocytes were drastically decreased *in vitro*. Monocytes displayed rapid cell death in the presence of nitric oxide (NO) and free Hb. An estimated 3~4 *μ*M NO, produced by 100 million of platelets, along with purified Hb (3 *μ*M) has decreased significantly the number of nonclassical monocytes than a classical subset, suggesting further the role of NO and free Hb (or Hb-activated platelets) in predisposing particularly a nonclassical monocyte subset towards cell death under hemolytic environments. Further, our data from hemolytic patients also supported the above *in vitro* observations. The peripheral monocytes from patients with either PNH or SCD displayed a direct correlation between high NO levels and cell death marker 7-AAD. Therefore, our data together suggest that due to the immune surveillance nature, the nonclassical or patrolling monocytes frequently encounter Hb-activated platelets, free Hb, and NO in the circulation of hemolytic patients and are predisposed to die rapidly. Our findings thus suggest that after encountering Hb-activated platelets, the classical monocytes are transformed into inflammatory subsets [34] and the nonclassical monocytes are predisposed to die and abolished completely from the circulation of these hemolytic patients including PNH and SCD.

## Data Availability

The data used to support the findings of this study are included within the article.

## Supplementary Materials

Supplementary Materials Suppl Fig S1: phenotyping of monocyte subsets for the identification of nonclassical monocyte subset using CD14, CX3CR1, and CCR2 surface markers in hemolytic patients. Suppl Fig S2: assessment of cell death in nonmonocytic cells at 2, 24, and 48 h, after incubation with Hb and GSNO. Suppl Table 1: the clinical information (monocyte frequency, TLC, sex, age, and treatment) of patients and controls used in the present study.
