# Supplementary material for: Absence of Nonclassical Monocytes in Hemolytic Patients: Free Hb and NO-Mediated Mechanism
Source: J Immunol Res. 2019 Mar 27;2019:1409383. doi: 10.1155/2019/1409383 (PMC6458887; doi:10.1155/2019/1409383)
Supplement: Supplementary Materials — Suppl Fig S1: phenotyping of monocyte subsets for the identification of nonclassical monocyte subset using CD14, CX3CR1, and CCR2 surface markers in hemolytic patients. Suppl Fig S2: assessment of cell death in nonmonocytic cells at 2, 24, and 48 h, after incubation with Hb and GSNO. Suppl Table 1: the clinical information (monocyte frequency, TLC, sex, age, and treatment) of patients and controls used in the present study. [file 1409383.f1.pdf]

“Absence of non-classical monocytes in hemolytic patients: free-Hb and NO-mediated mechanism”

“ Singhal *et al* ”.

“ Supplementary Figures and Table”

Suppl Fig S1

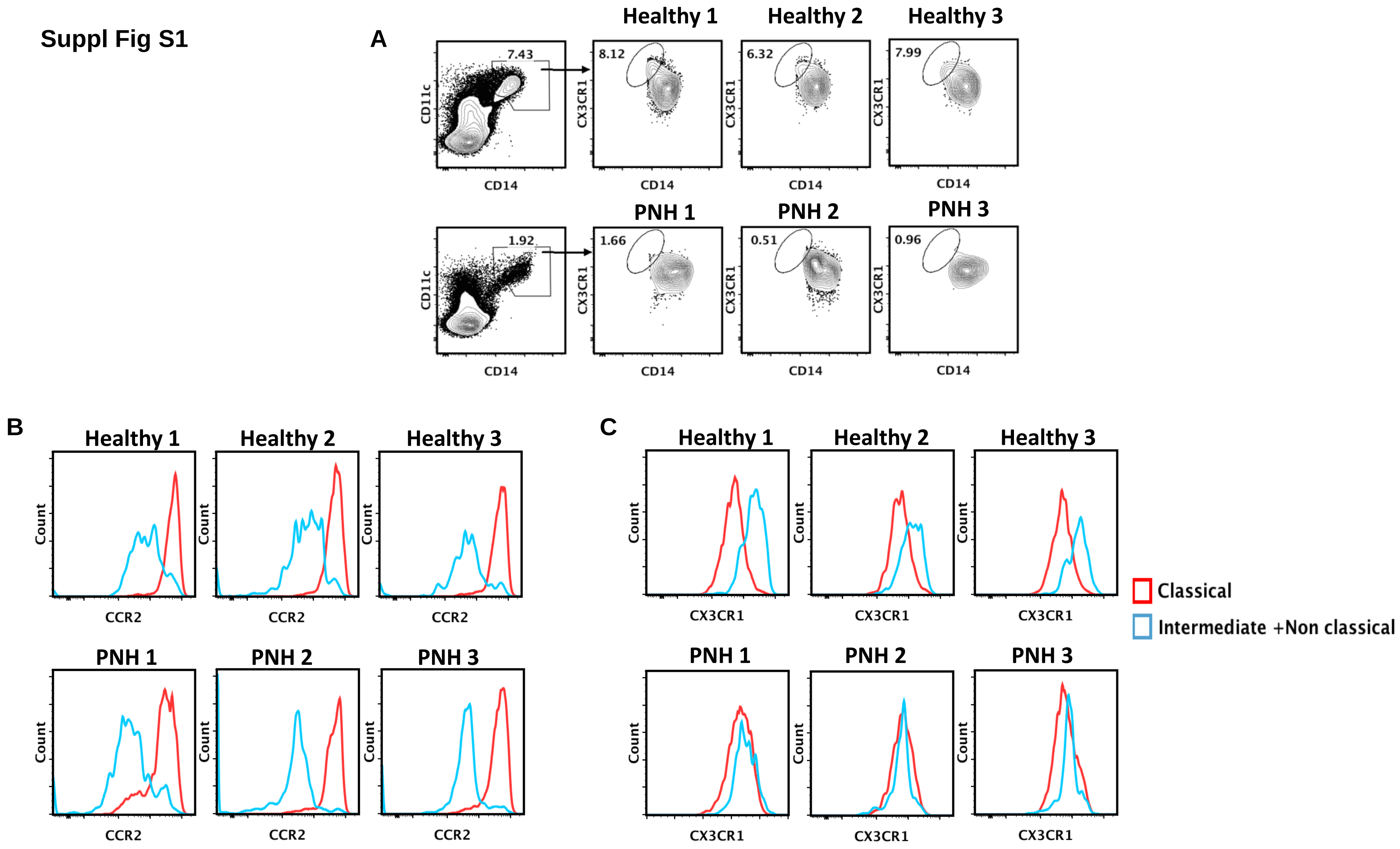

Supplement Figure S1. Immunophenotyping of monocyte subsets from hemolytic patients. (A) Representative FACS plots showing the gating strategy for the identification of non-classical monocyte subset using CX3CR1 and CD14. Total monocytes gated as CD11c^{+}CD14^{+} cells and non-classical subset was identified as CD14^{dim}CX3CR1^{high} cells in same healthy individuals (n=3) and PNH patients (n=3) mentioned in Fig. 5. Representative histograms showing expression levels of (B) CCR2 and (C) CX3CR1 on classical (red line) and intermediate + non-classical (blue line) subset. Classical, intermediate and non-classical cells were gated according to the strategy mentioned in Figure 1A

Suppl Fig S2

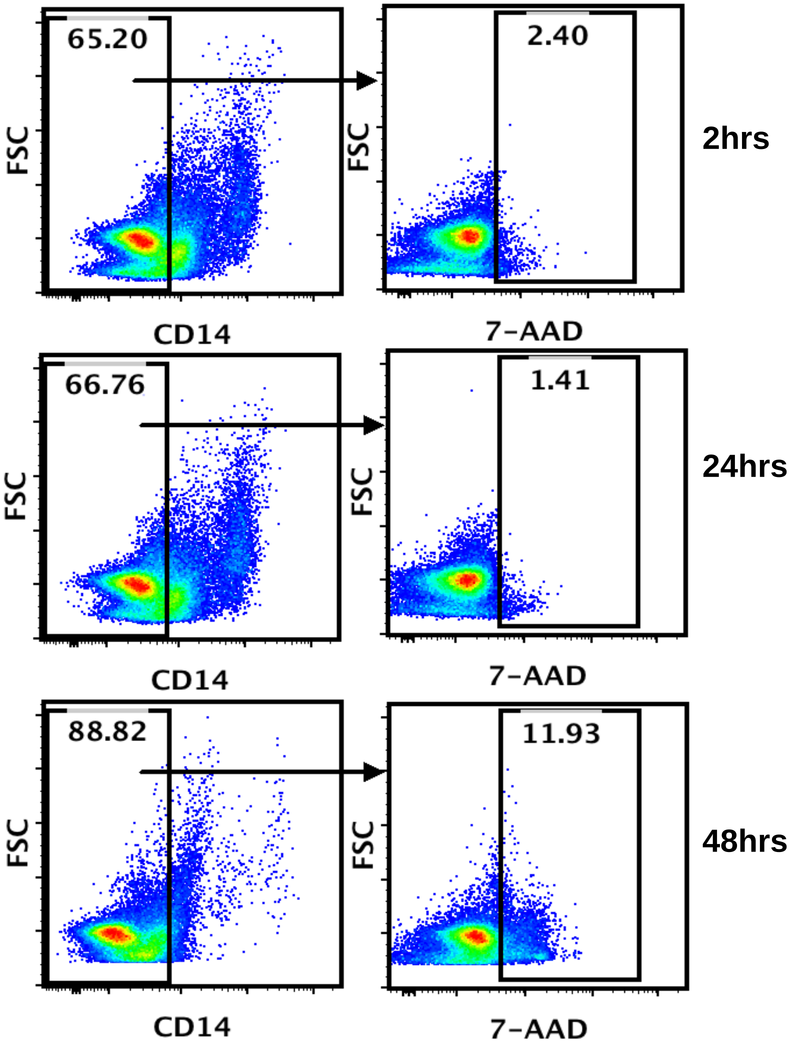

Supplement Figure S2. **Effects of Hb and NO on non-monocytic cells.** PBMCs isolated from healthy individuals were treated with Hb + GSNO for 2, 24 & 48 hrs. After incubation, cells were harvested and processed for 7-AAD staining to assess cell death by flow cytometry. $CD14^-$ cells gated as non-monocytic population (left column) and analyzed for 7-AAD staining (right column). Representative FACS plots from 3 independent experiments showing percentage of 7-AAD positive monocytes.

Suppl Table 1. Age, sex and leukocyte counts of participants

	**Healthy (n=20)**	**PNH (n=10)**	**SCD (n=10)**
**Sex, n (%)**			
**Male**	12 (60)	06 (60)	07 (70)
**Female**	08 (40)	04 (40)	03 (30)
**Age, mean (SD)**	31 (5)	30 (11)	24.5 (7.3)
**Total Leukocyte Counts, per µl of blood (SD)**	9253 (1527)	4529 (1399)	7071 (3141)
**Monocytes % (SD)**	10.88 (1.94)	6.10 (1.46)	4.75 (0.94)
**Treatments**	-	As per Physician	As per Physician
